# Circadian System and Melatonin Hormone: Risk Factors for Complications during Pregnancy

**DOI:** 10.1155/2015/825802

**Published:** 2015-03-02

**Authors:** F. J. Valenzuela, J. Vera, C. Venegas, F. Pino, C. Lagunas

**Affiliations:** ^1^Department of Basic Sciences, Universidad del Bío-Bío, Campus Fernando May, Avenida Andres Bello s/n, Chillán, Chile; ^2^Grupo de Ciencias Biotecnológicas, Basic Sciences Department, Universidad del Bío-Bío, Avenida Andres Bello s/n, Chillán, Chile

## Abstract

Pregnancy is a complex and well-regulated temporal event in which several steps are finely orchestrated including implantation, decidualization, placentation, and partum and any temporary alteration has serious effects on fetal and maternal health. Interestingly, alterations of circadian rhythms (i.e., shiftwork) have been correlated with increased risk of preterm delivery, intrauterine growth restriction, and preeclampsia. In the last few years evidence is accumulating that the placenta may have a functional circadian system and express the clock genes *Bmal1*, *Per1-2*, and *Clock*. On the other hand, there is evidence that the human placenta synthesizes melatonin, hormone involved in the regulation of the circadian system in other tissues. Moreover, is unknown the role of this local production of melatonin and whether this production have a circadian pattern. Available information indicates that melatonin induces in placenta the expression of antioxidant enzymes catalase and superoxide dismutase, prevents the injury produced by oxidative stress, and inhibits the expression of vascular endothelial growth factor (VEGF) a gene that in other tissues is controlled by clock genes. In this review we aim to analyze available information regarding clock genes and clock genes controlled genes such as VEGF and the possible role of melatonin synthesis in the placenta.

## 1. Introduction

Pregnancy is a complex and well-regulated temporal event in which several steps are finely orchestrated including implantation, decidualization, placentation, and partum [1]. The chronological transitions are critical for a normal pregnancy and any temporary alteration may have detrimental effects for fetal development and/or maternal health [2–4]. The placenta is the unit of communication and exchange between mother and fetus. This organ is in charge of bidirectional transference and metabolism of hormones, nutrients, and gases (oxygen/CO_2_) [5]. The major site of complication in pregnancy is the placenta and the main cause of development of obstetric syndrome is the placentation [6]. The impaired placentation causes spontaneous abortion, preeclampsia, preterm birth, and placental abruption [7]. Moreover, placenta mediates the maternal-fetal interaction in the regulation of glucocorticoids, human placental lactogen (hPL), human chorionic gonadotropin (hCG), and progesterone and estriol, among others [5, 8]. In this regard, hormonal production and activity is regulated by a circadian system, which, in fact, is composed by a family of genes named “clock genes” (Bmal1, Clock, Per1-3, and Cry1-2) [9].

## 2. Circadian Rhythms and Pregnancy

The circadian time-keeping system is actively engaged in the maintenance of normal physiology, not only in adults, but also during development [10]. Within an individual, the peak and trough of the rhythms for different physiological variables occurs at different clock times. For instance, in humans under normal light-dark condition, cortisol peaks at 08 h, while temperature peaks at 14–17 h, and melatonin at 02 h [11]. Similarly, during normal pregnancy different circadian rhythm are observed in the mother such as temperature [12, 13], leukocytes count, blood pressure [13], circadian pattern of weight gain [14], rhythms of uterine contraction, blood flow [15], and intra-amniotic fluid pressure [5, 15]. The final output of the circadian system during pregnancy is the labor. Humans and monkeys (diurnal animals) show a peak in the second middle of the night and early in the morning [3, 16, 17]. The rat and mice (nocturnal animals) show a time birth in the afternoon or final hours of the day [18, 19]. An important factor during the pregnancy is photoperiod, and light exposition during night hours (inhibition of melatonin production) is able to modify the hours of labor in monkeys and rats [18, 20]. At level of fetus, circadian rhythms of fetal heart rate and tachycardia are observed in twin pregnancy, showing a peak during light hours [21], showing that both the mother and the fetus have circadian rhythms.

Placenta during the pregnancy has important function of being in charge of bidirectional transference and metabolism of hormones, nutrients, and gases (oxygen/CO_2_) [5]. Some hormones produced by placenta show a circadian rhythm such as human chorionic gonadotropin (hCG) showing a peak at 12–15 h [8, 22, 23]. Progesterone and the products of aromatase from placenta which convert dehydroepiandrosterone sulphate (DHES) to estriol and estradiol [5] and placental lactogen show a circadian pattern in junctional and labyrinthine zones in the rat placenta [24].

The phase relation between the circadian rhythms of different physiological variables in the 24 h cycles generates an internal temporal order [25, 26], and recent data show that alterations of circadian rhythms correlated to increased susceptibility to cancer in humans [27]. In addition, it has been reported that incidence of breast cancer increases significantly in women working in shifts, being higher among individuals who spend more years and hours per week working at night [28]. In this sense, during human pregnancy several reports suggest alterations of circadian rhythms are correlated to increased susceptibility to pregnancy disease and a meta-analysis published by Bonzini et al. (2011) showed the impacts of shiftwork in those women [4]. Thus, shiftwork was associated with an increased risk of small for gestational age (<10th percentile) and low birth weight and reported eleven studies showing elevated risk for preterm birth [4]. In animal, pregnant rats exposed to light-dark cycle that mimics shiftwork showed an increase of fat weight and changes in peak hours of plasmatic glucose and leptine in three-month-old offspring [29].

**Figure 1:**
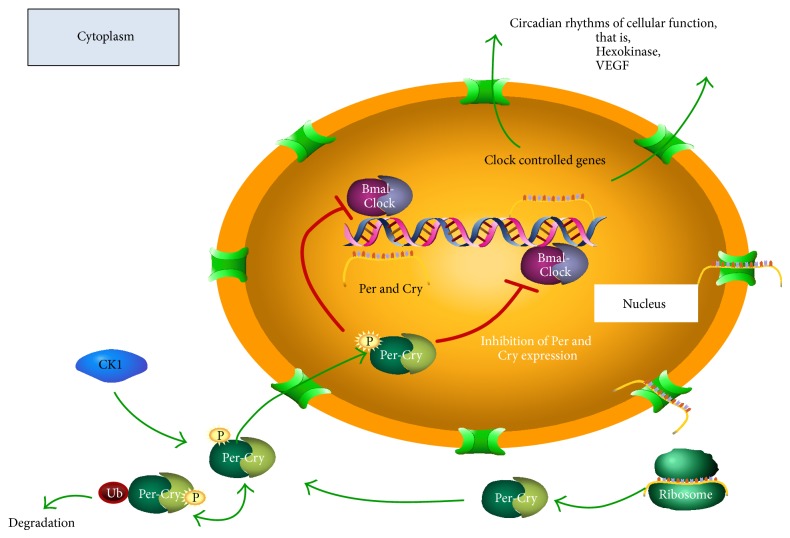
Molecular Circuit of Circadian Oscillator. See details in the text. Positive regulation of clock genes Bmal1 and Clock stimulate promoter of negative regulators Per 1-2, Cry 1-2 and controlled clock genes Hexokinase and VEGF.

In mammals, circadian rhythms are commanded by a central clock located in the Suprachiasmatic Nucleus of the Hypothalamus (SCN) acting on peripheral circadian clocks located in almost every tissue of the body, for example, in the adrenal gland [30]. In both the SCN and peripheral tissues, the circadian oscillation depends on a transcription/translation feedback loop of a group of genes collectively named “clock genes.” This family of clock genes include the transcription factors BMAL1 and CLOCK; the proteins encoded by genes* Per*1-3,* Cry*1-2 and the enzyme casein kinase 1 epsilon (CK1*ε*) [31] (see Figure 1). The mutation of any of the clock genes causes severe disruptions in circadian rhythms [32]. The heterodimer composed of CLOCK-BMAL1 protein is a positive regulator and binds to the E-box sequences (CACGTG) of the promoters of* Per* and* Cry*, inducing their expression. The negative regulator is a complex of the proteins PER and CRY which translocate to the nucleus and by protein-protein interaction with CLOCK-BMAL1 inhibits the transcription of* Per* and* Cry*. Translocation to the nucleus PER and CRY requires the formation of a complex with CK1*ε* and provides a delay in the system to achieve a period of 24 h [33]. Clock genes are expressed in multiple tissues: heart, liver, kidney, pancreas, muscle,* pars tuberalis*, adrenal gland, and isolated cells such as fibroblasts and cardiomyocytes [30, 34–41].

Circadian clock genes are expressed in the placenta of rats and mice [42, 43] and in the cell line of human trophoblast [44] previously stimulated by serum shock, a potent stimulator of the circadian system such as what has been described in fibroblast [45], immortalized human breast epithelial cell [46], or hepatoma cells [47]. The circadian expression of two genes potentially controlled by clock genes has been also shown in the placenta, the vascular endothelial growth factor (VEGF), and placental lactogen (PL-II). Thus, in the cell line of human trophoblast stimulated with serum shock, the VEGF is expressed with a circadian pattern [44]. Besides in culture of rat placenta stimulated by serum shock, a circadian rhythm of PL-II expression reaching a peak at 04:00 hrs in the junctional zone and at 16:00 hrs in labyrinth zones has been showed [24].

In humans, alterations in the levels of VEGF and human PL (hPL-II) proteins have been proposed as risk markers for preeclampsia or placental dysfunction [48–50] and we speculate that the chronodisruption might be part of pathophysiological process during pregnancies diseases. The vascular endothelial growth factor A (VEGF-A) has been related with occurrence of pregnancy pathologies such as preeclampsia [48, 51]. VEGF-A is a protein that is under control of the complex CLOCK-BMAL. Thus, the promoter of VEGF has four putative E-box elements (CANNTG) to respond to clock genes, showing a circadian pattern of expression in implanted tumor cell with a peak during light hours [52]. The* in vitro* transcription of VEGF cotransfected with CLOCK-BMAL increases the level of VEGF protein [52]. On the other hand, the transient expression of Per2 and Cry1 inhibits the expression of VEGF [52].* In vivo* experiments have showed that implanted tumor cells in mice are subordinated to SCN of the host animal. These cells show circadian rhythms of expression of clock genes and VEGF, observing a peak for the latter during the light hours, in a pattern similar to that observed for Bmal1 [52]. Considering that both reduced expression and activity of VEGF in the placenta [51] and altered circadian rhythms are associated with pathologies of pregnancy [4, 53], we could speculate that the circadian expression of clock genes would be controlling many placental functions in both normal and pathological placenta. This assumption would be difficult to test in human being; therefore investigation should include culture cell or animal models.

Nevertheless, an important question is whether in the culture of fresh human placenta, the clock gene expression is maintained as* in vivo* condition (i.e., circadian peripheral oscillator). In regard, we demonstrated the expression of the clock genes Bmal1 and Per2 during 36 hours in explant cultures of the adrenal gland without stimulation of serum shock, suggesting that the adrenal gland is a peripheral oscillator [30]. Whether the placenta contains a peripheral clock able to sustain an oscillation* in vitro* in absence of synchronizing stimuli is unknown. However, Frigato et al. (2009) showed that the circadian expression of Per2 is stimulated by serum shock in culture of trophoblast [44], suggesting that placental cell in culture might maintain the circadian oscillation observed* in vivo*.

*In vivo*, oscillation of clock genes in some peripheral organs requires the SCN. Guo et al. (2006) demonstrated in hamsters that oscillatory expression of the clock genes Per1, Per2, and Bmal1 in the heart, liver, kidney, renal cortex, adrenal medulla, muscle, and spleen is eliminated by ablating the SCN [54]. Transplantation of SCN restored the circadian oscillation of clock genes only in the liver and kidney, suggesting that the synchronizing signal from the SCN to the other organs involves neural pathways. These could include the autonomic nervous system or corticosterone and melatonin rhythms that are not restored by SCN transplants [54]. Since the placenta is not innervated but expresses melatonin receptors [55], we believe that melatonin could regulate the expression of clock genes in the placenta.

## 3. Melatonin and the Placenta

The pineal gland synthesizes melatonin, a lipophilic indoleamine hormone, increasing immediately in response to light-off. This increase gives chronobiotic information to the body for its circadian organization [11]. The extension of melatonin during the night is directly proportional to photoperiod (winter or summer), which in turn may modulate the gonadotropin axis and the time of mating sheep [35, 56]. A second function proposed for melatonin is as homeostatic hormone, regulating several aspects of fetal physiology. For instance, during the pregnancy, maternal melatonin provides a chronobiotic signal for the fetus and also plays a role in the development and maintenance of the fetal adrenal function under conditions suitable for fetal life, an effect that may also involve other fetal systems as shown in fetal sheep [9]. A third function of melatonin in placenta is to modulate the redox status, via both direct scavenger activity for radical species such as hydroxyl, alkoxyl, peroxyl, and nitric oxide (NO) [11, 57, 58], or regulating the expression of antioxidant enzymes such as catalase and manganese superoxide dismutase [59]. Additionally, in adult tissues melatonin has been shown to increase the expression of several genes such as Bax, p53, p21-27, caspases 3, 8, and 9 [60], NeuroD1, Pbef/Nampt, Hif1*α* [61], SGK, Nf*κ*bia, DNA-damage-inducible transcript 4, C/EBP-*δ*, pdk4, Ets-1, HSP [62], HOXA4, FOXO1A, GTFIIF1, PPAR*δ*, and TCEB3 [63] or decrease the expression of genes Bax, Bcl-2 [64]; P450 7*α* (CYP7B) [65]; metalloproteinase 9,3 (MMP-9, MMP-3) [66]; heat shock proteins HSP [62]; ZNF33A and PHF15 [63]. Moreover, we have found that melatonin inhibited the expression of Per1, Bmal1, and PGC1*α* in response to adrenocorticotropin (ACTH) in humans and sheep [38, 67], strongly suggesting a loop of regulation between melatonin production and clock genes expression.

**Figure 2:**
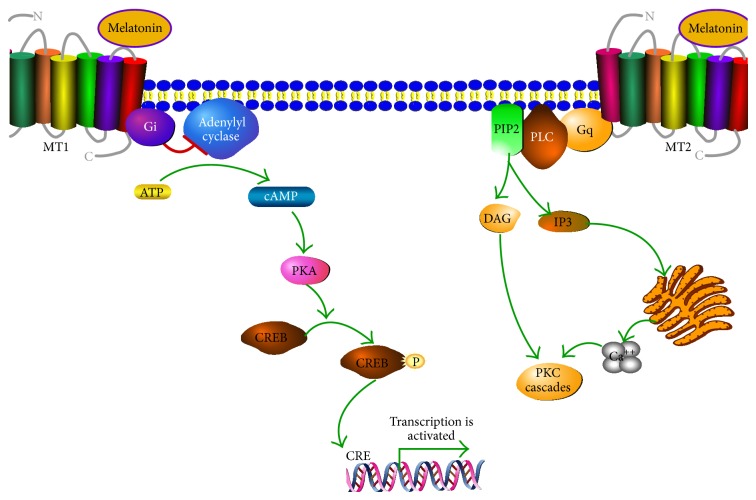
Signaling of MT1 and MT2. Both are G protein-coupled receptors. MT1 is associated with Gi protein and inhibition of adenylyl cyclase. MT2 receptor is associated with PKC stimulation and increase of calcium associated with IP3 (for details see Dubocovich et al. [11]).

Melatonin acts through membrane receptors (see Figure 2), although some effects of melatonin could be mediated by binding to endogenous ligand, the orphan nuclear hormone receptor superfamily RZR/ROR [68]. There are two melatonin membrane receptors named MT1 and MT2. Both are G protein-coupled receptors. Thus, MT1 is associated with (i) Gi and inhibition of adenylyl cyclase with decrease of cAMP, (ii) stimulation of potassium channels, and (iii) increase of Ca^2+^ via phospholipase C (PLC), whereas the MT2 receptor is associated with (a) Protein Kinase C (PKC) stimulation or (b) increase of calcium associated with IP_3_. Melatonin receptors MT1 and MT2 are present in many tissues [11, 69] although their function is not quite understood. However, their participation has been described in several mechanisms as indicated below.

### 3.1. Chronobiotic and Homeostatic Effects of Melatonin

*In vivo* treatment (2 hours) with melatonin increases the expression of Cry-2 in rat* pars tuberalis* [36] and decreases in the amplitude of the peak of Per1, suggesting an inhibitory effect of melatonin on this gene expression. In sheep, in which the secretion of melatonin was abolished by exposure to continuous light, expression of the clock genes Per 1-2 and Bmal1 continued in the* pars tuberalis.* However, melatonin treatment at any point in the day for a period of 3 hours induced mRNA expression of Cry-1 and inhibited the expression of Per 1-2 and Bmal1, effects that were not observed in the SCN [70]. In capuchin monkey, adrenal explants maintain an oscillatory expression of Bmal1 and Per2 for at least 36 hours in culture, and the treatment with melatonin decreased the expression of Bmal1 and Per2 [30]. Similar effects have been shown in the rat fetal adrenal in culture [40] and recently we detected that melatonin inhibit the expression of Per1 and Bmal1 in response to ACTH in newborn sheep and human adrenal gland [38, 67]. Moreover, melatonin via melatonin receptor MT1 and MT2 can modify the levels of pro- or antiapoptotic proteins such as Bax and Bcl-2 in human neuroblastoma cells [64]; similarly in placenta, treatment with 10 *μ*M of melatonin in villous trophoblast cells increases the survival via inhibition of loss of mitochondrial membrane potential and stimulating the formation of complex Bax/Bcl-2 (intrinsic via), expression of caspase-9, and the activation of ROCK1 [71].

### 3.2. Antioxidant Effects of Melatonin

Melatonin has a potent scavenger activity over hydroxyl, alkoxyl, and peroxyl radicals, as well as over species derived from nitrogen such as nitric oxide (NO) radicals [11, 57, 58]. In this regard, Milczarek et al. (2010) reported in placentas obtained after delivery a potent antioxidant effect of melatonin, specifically preventing the NADPH and iron dependent lipid peroxidation in the mitochondria [72]. Moreover, in studies of fetal growth restriction in animal model by placental ischemia, increased placental level of 8-hydroxy-2-deoxyguanosine has been detected (8-OHdG, i.e., a marker of a marker of oxidative DNA damage) [73] compared with controls. Both growth restriction and DNA damage were reverted when the rats received an oral dose of melatonin. Similarly, in rat undernourished pregnancy at day 20 of gestation, the fetal biometry showed lower values for fetal body weight and fetal body/placental weight ratio; moreover a tendency to a higher value of melatonin in maternal and fetal plasma is observed. Placentas from undernourished pregnancy showed no changes in the expression of antioxidant enzymes Mn-SOD, catalase, and GPx-1. However, the melatonin treatment during the pregnancy restores the placental efficiency at level of fetal body weight and fetal body/placental weight and induces the protein expression of Mn-SOD and catalase [59], suggesting that melatonin could be a candidate for protection/treatment of diseases characterized by placental ischemia such as intrauterine growth restriction, preeclampsia [73], or undernourished pregnancy [59]. Indeed, recent evidences have described that melatonin administration improved fetal-placental hemodynamic [74] and increased umbilical blood flow, an effect associated with “NO-dependent mechanisms” [75], as what occurs in cotyledonary placental arteries, via increased sensitivity to vasorelaxation agents such as bradykinin and lower contractile response to noradrenaline [76]. Additionally, melatonin administration reverted the increment of lipid peroxidation in the placenta and liver of mother and fetus exposed to cholestasis of pregnancy [77]. Interestingly, in human it has been described that oral melatonin administration increased glutathione peroxidase (GSH-Px) in the placenta of Japanese women with pregnancies of 7 and 9 weeks of gestation [78].

Protective effects of other antioxidant agents on fetal growth and development strongly support the protective effects of melatonin in adverse pregnancy being due to its antioxidant rather than antioxidant-independent properties, for example, developmental programming of cardiovascular dysfunction by prenatal hypoxia and oxidative stress.

### 3.3. Melatonin and Vascular Remodeling

In rats, it has been described that melatonin regulate the levels of NO and VEGF in the nervous system, that is, in the choroid plexus, cerebellum, periventricular white matter, and hippocampus [79–82]. Moreover, melatonin treatment for short or long periods of time inhibits the endogenous expression of VEGF and hypoxia induced factor 1 alpha (HIF-1*α*) in tumor cells [83]. In addition, melatonin induces the expression of VEGF and matrix metalloproteinase- (MMP-) 2 in extrapineal tissues such as gastric mucosa [66]. Also melatonin increases bone defect repair in rabbits [84]. All these indirect evidences suggest that melatonin may control vessel formation. Nevertheless, other reports have showed reduced tumor angiogenesis in mice treated with melatonin [85], as well as reduced human umbilical vein endothelial cell proliferation/migration induced by VEGF [86, 87].

## 4. Extrapineal Production of Melatonin: The Placenta

The critical enzymes for the synthesis of melatonin are arylalkylamine N-acetyltransferase (AA-NAT) and hydroxyindole O-methyltransferase (HIOMT). These enzymes are expressed in the major site of synthesis of melatonin, in the pineal gland with a circadian pattern of activity for AA-NAT [11], and in the human placenta [88]. Therefore, the human placenta can be considered as an extrapineal source of melatonin similar to retina [89] and lymphocytes [90]. In this regard, expression (mRNA and protein) of AA-NAT and HIOMT has been detected in both cell line of human placental trophoblasts and human term placentas [71, 88, 91]. In addition, other reports have described the presence of MT1 and MT2 in total human placenta [55] or placental endothelial cells [88]. Interestingly, MT1 is expressed at high levels in the junctional zone during day hours (16:00 hrs) and in the labyrinth during night hours (04:00 hrs) in mice [24].

We do not know whether the production of melatonin in the human placenta changes with the hours of the day. Interestingly, in normal pregnancies, serum levels of melatonin increase progressively until 32 weeks of gestation and decrease prior to delivery reaching the lowest levels at 2 days postpartum [92–95]. Moreover, the level of melatonin is higher in human twins pregnancies [92], as well as it is correlated with the number of pups in animal models [94], suggesting a relationship between placental volume and melatonin level. Nevertheless, in human pregnancies associated with placental alteration, the maternal circadian production of melatonin is lost and it is associated with diminished levels of melatonin. For example, circadian alteration has been observed in humans at the level of diastolic blood pressure, plasma concentration, and circadian production of melatonin during preeclampsia. After pregnancy, these women showed a normal circadian diastolic blood pressure but maintained altered rhythm for melatonin [53]. In contrast to humans, AA-NAT is not expressed in the placenta of rats, and an increase of maternal melatonin is a consequence of placental factor released into the circulation, which would stimulate the maternal pineal gland [94].

**Figure 3:**
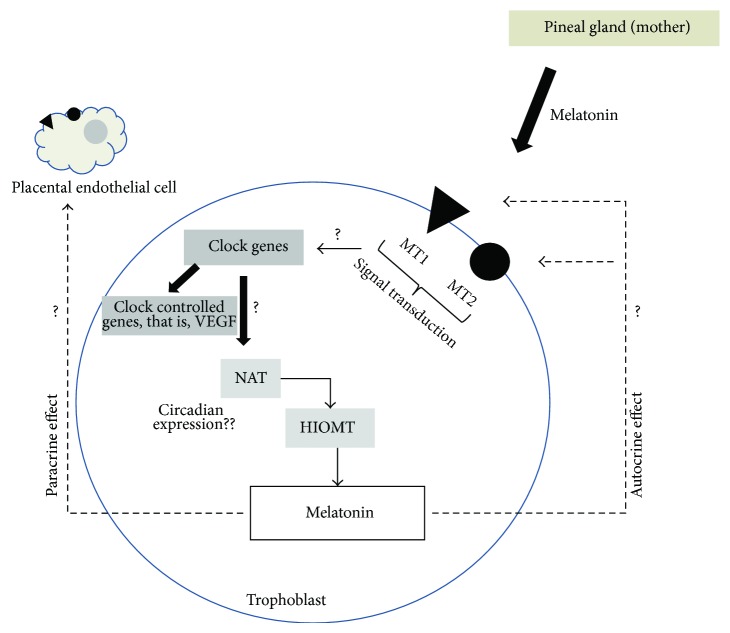
Autocrine and paracrine effects of melatonin over circadian system and enzyme of synthesis of melatonin localized in trophoblast and endothelial cells.

Although melatonin has multiple effects on placental function, including induction of the expression in undernourished pregnancy of antioxidant enzymes such as superoxide dismutase (Mn-SOD) and catalase [59], prevention of oxidative stress-mediated injury during placental ischemia [73], inhibition of hCG release in trophoblast cells [91], and inhibition of formation of proapoptotic complex [71], the role of this local production in the human placenta is not well understood and opens the possibility of autocrine, intracrine, or paracrine effects. Although there are no direct evidences, other studies using lymphoid cells, which express the enzymes NAT and HIOMT and produce 5-fold more melatonin than pineal gland, have described that melatonin produced locally has a minor effect on melatonin serum level but has a local role incrementing the IL-2 production [90]. This last effect was inhibited by luzindole and CGP 55644, an antagonist of membrane and nuclear receptors of melatonin [96]. Then, it is feasible that melatonin can be modulating the circadian system in the placenta, see Figure 3, producing changes in clock genes that may control VEGF or enzymes NAT and HIOMT. We encourage the scientific community with this idea.

## 5. Conclusion

Participation of the circadian regulatory system has been described as a feedback regulatory loop where melatonin is downregulating the clock genes. In turn, clock genes upregulate the expression of output genes such as VEGF, SOD, NAT, and HIOMT. Current evidences describe that the placenta is a nonpineal organ, which synthesizes melatonin, and the activity of this organ is regulated by a circadian system. Moreover, an impaired circadian system is associated with an altered production of melatonin; however, the effect of this alteration on clock gene expression or output genes (VEGF) has not been described. We believe that circadian system and melatonin are a keystone molecule in the placental physiology, but more studies are necessary in order to test this idea. In this regard, we propose that melatonin may control clock gene expression (Bmal1, Per1-3, Cry1-2, and Clock) and output genes (VEGF) during normal pregnancies.

## Glossary

BMAL1: Aryl hydrocarbon receptor nuclear translocator-like
CLOCK: Circadian locomotor output cycles kaput
PER: Homolog of period,* Drosophila*
E-BOX: Promoter sequence for binding of clock-bmal1 complex (CACGTG)
CREB: cAMP response element-binding protein
PGC1*α*: Peroxisome proliferator-activated receptor-gamma, coactivator 1*α*.
